# Age period cohort analysis of chewing ability in Korea from 2007 to 2018

**DOI:** 10.1038/s41598-021-94086-8

**Published:** 2021-07-19

**Authors:** Nam-Hee Kim, Ichiro Kawachi

**Affiliations:** 1 Department of Dental Hygiene, Wonju College of Medicine, Yonsei University, 20 Ilsan-ro, Wonju, Gangwon-do 26426 Republic of Korea; 2 Department of Social and Behavioral Sciences, Harvard T.H. Chan School of Public Health, Boston, MA 02115 USA; 3 Department of Dental Hygiene, College of Software and Digital Healthcare Convergence, Mirae Campus, Yonsei University, Wonju, Gangwon-do 26493 Republic of Korea

**Keywords:** Environmental social sciences, Medical research, Risk factors

## Abstract

There have been marked improvements in oral health in Korea during the past 10 years, including chewing ability. We sought to disentangle age, period, and cohort effects in chewing ability between 2007 and 2018. We analyzed data from the Korea National Health and Nutrition Examination Survey. The main variable was chewing difficulty, which was assessed among participants aged 20 years and older. APC analysis revealed three trends in chewing difficulty: (1) there was an increase in chewing difficulty starting at around 60 years of age (age effect), (2) there was a steady decrease in chewing difficulty during the observation period (period effect), and (3) chewing ability improved with each successive generation born after 1951 (cohort effect). Regarding recent improvements in chewing ability, cohort effects were somewhat more important than period effects.

## Introduction

Recent studies have suggested that oral health has been improving in many countries^1–9^, with decreased incidence of dental diseases and increased accessibility of dental care. Contributory factors could include improved proximal determinants of oral health (i.e., health behaviors and sugar consumption), as well as improving structural determinants (i.e., socioeconomic context and environmental context) over several decades.

Oral health is affected by exposures throughout the entire life course, beginning in the prenatal period, and is strongly socially patterned^10^. Studies have tried to identify age, period, and cohort effects to clarify the impact of social and environmental exposure and thereby find compelling evidence of the importance of preventive factors in oral health^1,11–19^. Cohort effects on oral cancer mortality have been revealed in two studies, supporting common risk factors theory^12,15^. These studies suggested that the influence of smoking and alcohol consumption contributed to marked changes in the cohort-effect curvature^15^. Secular changes in oral health behaviors (such as regular brushing, use of fluoridated toothpaste) as well as changes in dental practices may result in cohort effects on oral health outcomes over time (i.e., dental care utilization and number of remaining teeth)^17^. Dental insurance typically has a period effect, i.e., covering it causes changes in access before and after the policy. However, it could influence both period and cohort effects, in terms of a trend changing from problems in visiting patterns to routine and maintenance care^1^.

In the Korean context, the national dental sealant project launched in 2000 contributed to the prevention of dental caries (a period effect)^4^. For example, dental sealant use increased (24.8% to 48.9%), while untreated dental caries decreased (49.7% to 19.8%) from 2003 to 2010 among 12-year-olds. Further, the filled teeth for dental decay and the number of teeth increased in the recent birth cohorts (a cohort effect)^18^. However, evidence has less focused on subjective oral health outcomes which more likely to need-based predict for the population oral health^20,21^.

Chewing ability is associated with accumulated lifetime impacts on oral health; that is, it is not just an issue for older persons. Chewing ability is associated with the number of teeth affected by dental caries, periodontal disease, and inadequate access to dental care. In turn, aspects of oral health can influence social outcomes (i.e., job opportunity and economic activity)^22,23^ as well as health outcomes (i.e., life expectancy)^24^. Recent evidence suggests that improving trends in chewing ability might be attributed to external changes beyond dental treatment interventions^25^. This is because chewing ability has improved in both older and younger adults, which implies the need to identify age- and period-cohort patterns in these trends.

An age effect on chewing ability implies that there are changes in oral health as a result of accumulated physiological changes over time—for example, the effects of aging on periodontal tissues based on molecular changes in periodontal cells which intensify bone loss in elderly patients with periodontitis^26^. If improved chewing ability is due to a period effect, then the current trends may persist into the next generation since the same exposure factors would affect all ages simultaneously. If the chewing ability pattern is due to a birth cohort effect, then this will contribute to an improvement in oral health in the future because South Korea is advancing social determinants of oral health.

Thus, we sought to identify age, period, and cohort effects related to trends in improved chewing ability, to reflect overall oral function for persons of all ages.

## Methods

### Ethics approval and consent to participate

This study used open access data from the Korea National Health and Nutrition Examination Survey (KNHANES) for 2017 and 2016–2018 conducted by the Korea Centers for Disease Control and Prevention (KCDC). All participants of the KNHANES provided written informed consent to participate in the survey. The KNHANES was approved by the Institutional Review Board (IRB) of the KCDC. This is a publicly available, secondary dataset. Our institute determined that the use of the KNHANES dataset does not meet the criteria for human subject research, and was therefore exempt from IRB approval. We confirmed that all methods were performed in accordance with the relevant guidelines and regulations.

### Data sources

We used the Korea National Health and Nutrition Examination Survey (KNHANES) data from 2007 to 2016–2018. The KNHANES is a nationally representative cross-sectional survey conducted by the Korea Centers for Disease Control and Prevention (KCDC). The survey includes approximately 10,000 individuals aged 1 year or over during each survey year. Among participants, less than 3% were excluded because of missing values for our variables of interest (i.e., chewing difficulty, age, sex, and study year). Chewing difficulty is defined as difficulty or discomfort during mastication. It was assessed using self-responses to the following in-person questions: ‘Do you have difficulty or discomfort when chewing food due to oral problems, including teeth, dentures, or gums?’ and ‘If you use dentures, please describe your experience of wearing dentures’. Individual responses of experiencing ‘difficulty’ and ‘severe difficulty’ were categorized as ‘chewing difficulties’ and assigned a value of 1; whereas responses of ‘no difficulty’, ‘little difficulty’, and ‘some difficulty’ were categorized as ‘no chewing difficulties’ and assigned a value of 0. Age-adjusted prevalence of chewing difficulty was calculated using the standard population provided by the Korea National Statistical Office’s resident population registry^27^. In this analysis, age was the self-reported age of the respondents at the time of the survey interview. This ranged from the oldest respondents (80 years or older) to the youngest respondents (aged 20 years). Period represented the years of data collection (2007 to 2016–2018). Birth year was the age subtracted from the survey year. Birth cohort was categorized based on arbitrary 10-year periods from 1900 to 1992.

### Statistical analysis

We performed two-stage procedures to estimate age, period, and cohort effects and to confirm robustness of the analysis. First, we constructed age-period-cohort (APC) models (with “acp” parametrization) using the “apcfit” Stata command to apply constraints to overcome the identifiability issue of APC models. Natural splines were used to estimate each of the three effects, which were then combined to obtain estimated rates^28^. We used yearly interval knots each for age, period, and cohort variable to fit the model appropriately and to ensure sensitivity, which can be dictated by the width of the intervals for the age and period terms. Age, period, and cohort were treated as continuous variables. We also investigated the effect of gender as a covariate. We used a generalized linear model framework with a Poisson family error structure, a log link function, and an offset of log (person risk time).

Second, we used the “grmean” Stata command to plot standardized rates and to plot observed and fitted rates against another variable, with separate lines and symbols used for different groups from the APC models^29^.

Finally, we confirmed the APC model fit to possible combinations of age, period, and cohort effects using the Akaike information criterion and Bayesian information criterion^30^. We also conducted a sensitivity test to verify changes in the trends in age-adjusted rates using Joinpoint, which was developed by the Surveillance Research Program of the United States National Cancer Institute^31^.

We used Stata statistical software (Stata Corp. 2017. Stata Statistical Software: Release 15. College Station, TX: Stata Corp LLC.) for all statistical analyses.

## Results

**Figure 1:**
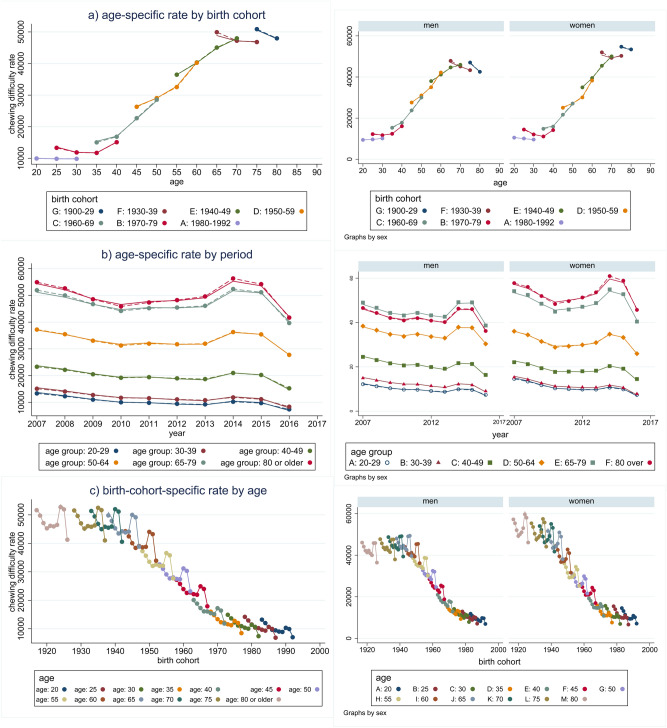
Age, period, and cohort effects on chewing difficulty. Chewing difficulty prevalence between 2007 and 2018 is shown overall, and for men and women: (**a**) age-specific difficulty by birth cohort overall, and for men and women, (**b**) by period overall, and for men and women, (**c**) birth-cohort-specific rate by age overall, and for men and women.

Figure 1 illustrates the results of the age, period, and cohort effects both genders combined, and men and women separately. The APC analysis revealed three trends in chewing difficulty: (1) an increase in chewing difficulty at around 60 years of age (age effect), (2) a steady decrease from 2007 to 2010 and an increase between 2010 and 2014, followed by a decline (period effect), and (3) greater difficulty in the generation born before 1951 compared to the generation born after 1951; chewing difficulty gradually decreased thereafter in each generation (cohort effect).

Although there were apparent differences between men and women in the three results, we confirmed that there was no gender effect on chewing difficulty across the entire dataset after adjusting for other effects in the APC model (Supplementary Table S1).

Table 1 shows the results for each fitted model. The full ACP and APC models, which had the lowest Akaike information criterion and Bayesian information criterion values, were considered the best fit out of the four models^32^. We confirmed that both the ACP and APC parameterizations represent an adequate fit of our data. Regarding the AIC statistics in Table S2 and Table S3, the only difference between ACP and APC specification is indicated in the 5th decimal place. We also confirmed that the BIC provided comparable results for the ACP and APC specifications.

**Table 1:** Goodness of fit of APC for chewing difficulty among Korean adults from 2007 to 2016–2018.

Model	AIC	BIC	df	Log likelihood	N
**Overall**					
APC	24,707.52	24,792.63	15	− 12,338.76	2151
ACP	24,707.51	24,792.61	15	− 12,338.75	
AC	25,471.14	25,533.55	11	− 12,724.57	
AP	24,727.62	24,790.03	11	− 12,352.81	
**Men**					
APC	10,881.66	10,956.3	15	− 5425.829	1071
ACP	10,881.65	10,956.3	15	− 5425.827	
AC	11,194.56	11,249.3	11	− 5586.282	
AP	10,885.88	10,940.62	11	− 5431.94	
**Women**					
APC	13,796.39	13,871.16	15	− 6883.194	1080
ACP	13,796.38	13,871.15	15	− 6883.190	
AC	14,249.61	14,304.45	11	− 7113.807	
AP	13,810.92	13,865.75	11	− 6894.458	

AIC: Akaike information criterion; BIC: Bayesian information criterion (BIC); d.f.: degree of freedom; APC: age-specific rates for a particular period (2007), after adjustment for cohort effect model; ACP: age-specific rates for a particular cohort (1951), after adjustment for period effect model; AC: age/cohort model; AP: age/period model.

**Figure 2:**
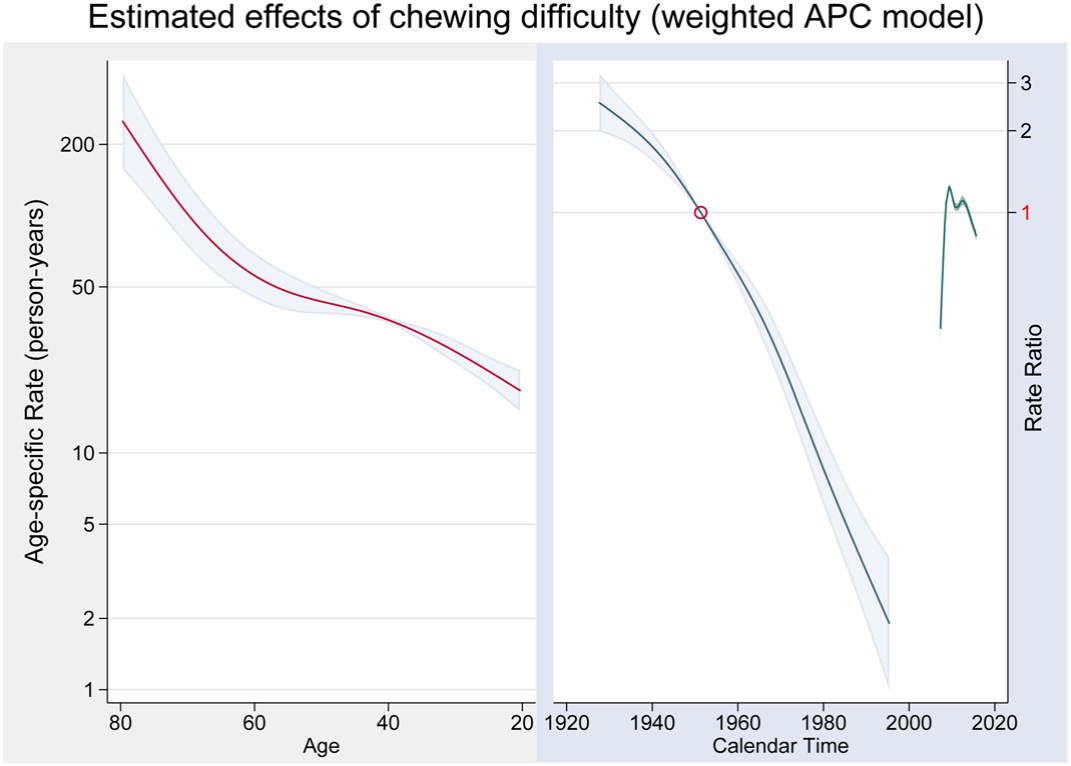
APC model estimation of chewing difficulty. Age-specific chewing difficulty, referenced to 1951. The regions surrounding the lines provide 95% confidence intervals.

Figure 2 shows the ratios obtained from the APC analysis with “acp” parametrization which can be interpreted as showing the longitudinal effect of age for the reference cohort and how this differs across cohorts^32^. It reveals age-specific rates for those born in 1951, after adjustment for the period effect. The left side indicates that among people born in 1951, chewing difficulty increased steadily until around 60 years of age, and then increased markedly thereafter. The right side shows that the cohort effect (including drift) decreased steadily until the 1990s. In the cohort analysis, those born in 1951 are the reference [= 1] and represent the point of acceleration of risk. The graph also shows a lower proportion of participants with chewing difficulty in later cohorts (including Baby Boomers) born between the 1960s and the 1990s. A longitudinal age effect also was confirmed, suggesting that the prevalence of chewing difficulty increases as the cohort ages. (Supplementary Figure S1).

**Figure 3:**
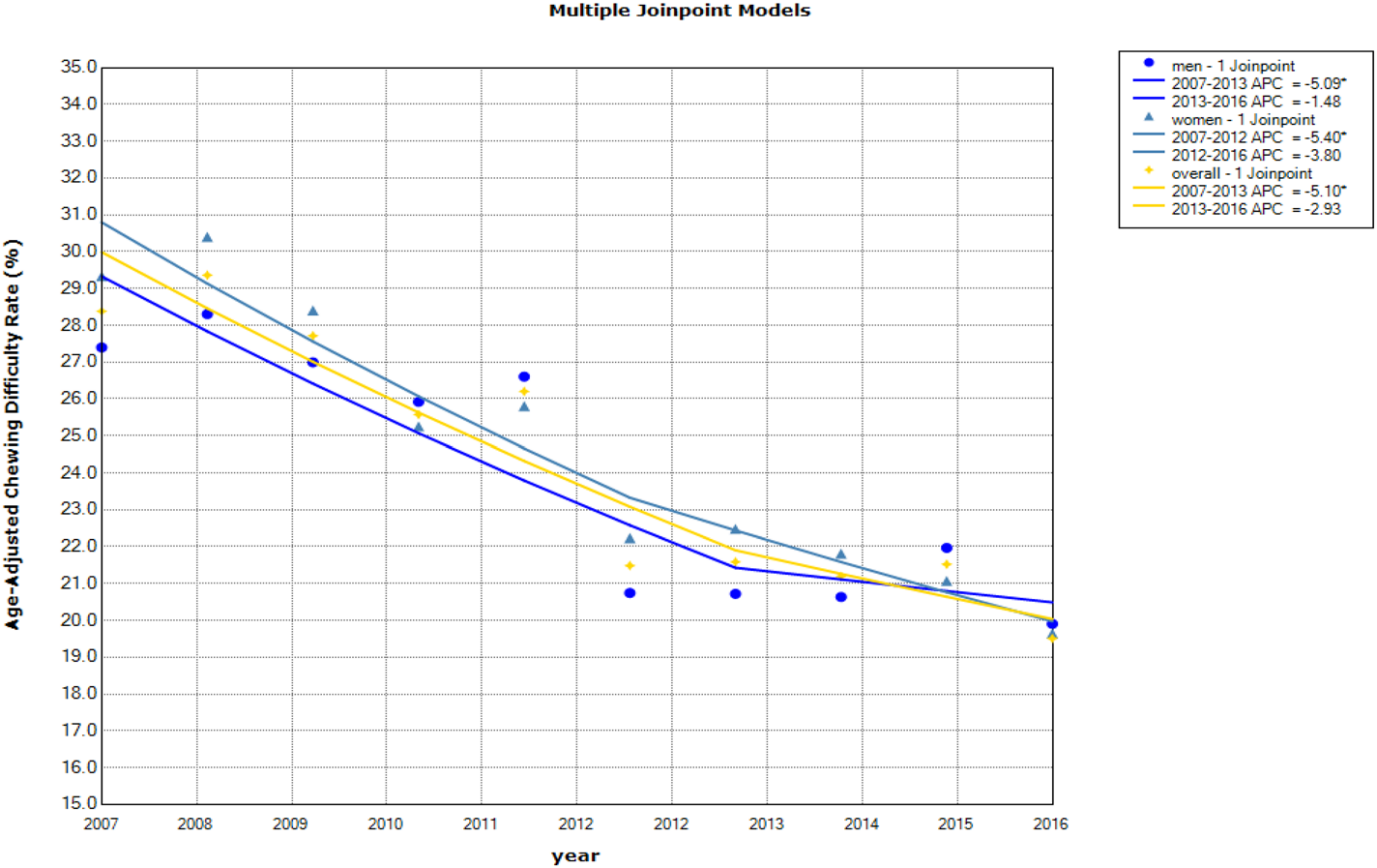
Multiple Joinpoint models of chewing difficulty. APC denotes annual percent change.

We confirmed the validity of our APC model. In the Joinpoint regression analysis, changes in the linear trends were detected. Chewing difficulty prevalence decreased drastically from 2007 to 2013 (annual percent change: APC: − 5.10, 95% CI − 8.5 to − 1.6) and decreased gradually from 2013 to 2016 (APC: − 2.9, 95% CI − 12.8 to 8.1). In men, the prevalence of chewing difficulty decreased significantly from 2007 to 2013 (APC: − 5.1, 95% CI − 9.5 to − 0.5) and decreased from 2013 to 2016 (APC: − 1.5, 95% CI − 14.4 to 13.3). In women, the prevalence of chewing difficulty decreased significantly from 2007 to 2012 (APC: − 5.4, 95% CI − 8.2 to − 2.5) and decreased from 2012 to 2016 (APC: − 3.8, 95% CI − 7.9 to 0.4; Fig. 3).

## Discussion

Our findings show that chewing difficulty trends are largely driven by a birth cohort pattern. The period effects suggest a very slight decline in chewing difficulty up to about 2010, followed by an unexpected (and unexplained) increase in chewing difficulty between 2010 and 2014. The cohort effect curve indicated declining rates of chewing difficulty in birth cohorts born after 1951.

Our findings suggest that social environments affect oral health outcomes, consistent with findings in other countries^1,17^. These results indicate that cohort succession may be the mechanism by which chewing ability has consistently shown an improvement over time in Korea^25^. As older cohorts—characterized by higher chewing difficulty—are replaced by younger cohorts, chewing ability improves overall. This suggests that older cohorts with poor oral health outcomes have been replaced by younger cohorts with better outcomes. Moreover, our findings are consistent with a cohort effect previously reported on population trends in the average number of teeth^18^.

To explain the mechanisms underlying the association between birth cohort and chewing ability, it may be helpful to consider social conditions at multiple levels which affect oral health outcomes^33^. At the broadest level, social environmental change (e.g., the nutrition transition^34–36^) may have contributed to improved chewing ability among those born after the 1951 cohort. Babies born just after the Korean War experienced a dramatic change in nutritional environment (e.g. exposure to candy, and sweets)^34,35^, which may have set them up for poor oral health for the rest of their lives. The same nutrition transition has been linked to a shift in disease patterns, away from infectious diseases toward chronic diseases such as diabetes, cardiovascular disease, and cancer^34^. High caries is associated with the availability of sugar and this often happens when a country is undergoing rapid economic transition^34,37,38^. For example, in Kenya, the prevalence of dental caries was low (comparable to Ethiopia and Senegal) when the mean daily per capita supply of sugar was 50 g or less^37^. Dental caries increased in parallel with sugar consumption^38^. However, for a brief period after a country becomes integrated with the world economy, there is a huge influx of cheap and affordable “junk foods” (i.e., candy and sugary drinks), which leads to deterioration in oral health^10,39^. The prevalence of dental caries dramatically increased up to 1995, followed by a decline in recent years. For example, the mean number of decayed, missing, filled teeth (DMFT) was 0.6 in 1972, 3.1 in 1995, and down again to 1.4 in 2013–2015 among 12-year-olds^3,40^. More recent generations may not have the same exposure, i.e., educated parents became more strict about allowing their children to eat sweets^4,41^.

Fluoride exposure may contribute to oral health later in adulthood^8,9,42,43^, Contemporaneous and dose–response correlations have been identified^42,43^. Unfortunately, we could not highlight cohort effects of fluoride exposure due to limited data. In Korea, approximately 13% of the national population has been treated; community water fluoridation was implemented in 1981 and extended until 2003^44^. Individuals could have potentially received exposure for up to 22 years (for those born in 1981). However, if this had a big effect, we would expect to find a dramatic decline in chewing difficulty for generations born after the late-1970s. But we could not find such an effect because the cohorts born after 1970 have not yet reached the age when people begin to develop chewing difficulty. Thus, further observation is warranted into the future.

We could not fully explain the period effect in our data, which appeared to show an unexpected increase in chewing difficulty between 2010 and 2014, before declining again (Fig. 1b). A period effect was identified in the APC model and confirmed using Joinpoint. That is, there was one slope change in the chewing difficulty trend. In addition, we confirmed changes in the linear trends against the alternative of one Joinpoint in the age-adjusted prevalence of chewing difficulty (Fig. 3). During this period, dental insurance coverage was expanded to include dental prosthetics treatment (starting in 2012) and dental scaling (starting in 2013). Older adults (aged 65 and older) are eligible for the former, while the latter is available for those aged 19 and over. However, it seems unlikely that these interventions could be responsible for changing oral health outcomes over the short time period^25,45,46^. Hence, our anomaly may be a measurement artefact^47^.

Some limitations should be considered when interpreting our findings. We could not consider the individual level factors that contributed to changes in chewing ability. Our analyses did not define birth cohort based on prior theory (i.e., Generation X) due to lack of evidence of their linkage to oral health. Another potential issue is that our age-effect shows that most people do not develop chewing difficulty until after age 60 because they still have enough teeth left. This means that we could not observe what happened to generations born after 1970, because they are still not old enough yet to develop chewing difficulty. It is possible that when the generation born after 1970 reach older ages, they will begin to show more chewing difficulty.

## Conclusions

Our study showed that chewing ability improved over the previous decade. Regarding recent improvements in chewing ability, cohort effects were somewhat more important than period effects.

## Supplementary Information

Supplementary Information.

## Data Availability

The data from the fourth KNHANES is open to the public, therefore, any researcher can obtain data upon request from https://knhanes.cdc.go.kr. The Korean National Health and Nutrition Examination Survey (KNHANES) data are publicly available through the KNHANES website (https://knhanes.cdc.go.kr/knhanes/eng/sub03/sub03_01.do).
