# Supplementary material for: Age period cohort analysis of chewing ability in Korea from 2007 to 2018
Source: Sci Rep. 2021 Jul 19;11:14660. doi: 10.1038/s41598-021-94086-8 (PMC8289934; doi:10.1038/s41598-021-94086-8)
Supplement: Supplementary file 1 — Supplementary Information. [file 41598_2021_94086_MOESM1_ESM.pdf]

Supplementary information

Age period cohort analysis of chewing ability in Korea from 2007 to 2018

Nam-Hee Kim[1,2,3*] & Ichiro Kawachi[2]

[1]Department of Dental Hygiene, Wonju College of Medicine, Yonsei University, Wonju, Gangwon-do, Republic of Korea, nami71@yonsei.ac.kr, nhkim@hsph.harvard.edu

[2]Department of Social and Behavioral Sciences, Harvard T.H. Chan School of Public Health, Boston, Massachusetts, ikawachi@hsph.harvard.edu

[3]Department of Dental Hygiene, College of Software and Digital Healthcare Convergence, Mirae Campus, Yonsei University, Wonju, Gangwon-do, Republic of Korea

***Corresponding Author:**

N.H. Kim, MPH, PhD

Department of Dental Hygiene, Wonju College of Medicine, Yonsei University, 20 Ilsan-ro, Wonju, Gangwon-do 26426, Republic of Korea

Telephone: +82-33-741-0391

Fax: +82-33-735-0391

E-mail: nami71@yonsei.ac.kr

ORCID 0000-0001-5463-0073

Supplementary Table S1. Proportional effect for gender of the APC model

. glm D _spA* _spP* _spC* sex, family(poisson) lnoffset(Y) nocons eform

Iteration 0: log likelihood = -12784.784
Iteration 1: log likelihood = -12339.056
Iteration 2: log likelihood = -12338.739
Iteration 3: log likelihood = -12338.739

Generalized linear models		No. of obs =	2,151
Optimization: ML		Residual df =	2,135
		Scale parameter =	1
Deviance =	20289.1613	(1/df) Deviance =	9.50312
Pearson =	16820.47918	(1/df) Pearson =	7.878445
Variance function: V(u) = u		[Poisson]	
Link function : g(u) = ln(u)		[Log]	
		AIC =	11.48744
Log likelihood =	-12338.73888	BIC =	3905.837

D	IRR	OIM Std. Err.	z	P>|z|	[95% Conf. Interval]	
**_spA1_intct**	**.5000548**	**.0330868**	**-10.47**	**0.000**	**.4392348**	**.5692965**
_spA2	.5334765	.0377076	-8.89	0.000	.4644618	.6127462
_spA3	.859501	.0348079	-3.74	0.000	.793916	.9305039
_spA4	1.090595	.0296348	3.19	0.001	1.034032	1.150253
_spA5	1.041916	.0202703	2.11	0.035	1.002935	1.082412
_spA6	1.001461	.0133681	0.11	0.913	.9755996	1.028008
_spP1	.8788591	.0080408	-14.11	0.000	.86324	.8947609
_spP2	1.266472	.0127993	23.38	0.000	1.241633	1.291809
_spP3	1.065535	.0091474	7.39	0.000	1.047757	1.083616
_spP4	.9903531	.0089857	-1.07	0.285	.9728971	1.008122
**_spC1_ldrft**	**.9382173**	**.0035513**	**-16.85**	**0.000**	**.9312827**	**.9452035**
_spC2	1.193815	.0458108	4.62	0.000	1.107321	1.287065
_spC3	1.007687	.024863	0.31	0.756	.9601156	1.057615
_spC4	1.001961	.0235428	0.08	0.934	.9568643	1.049183
_spC5	.979146	.0207196	-1.00	0.319	.9393671	1.02061
**sex**	**.9969846**	**.0172489**	**-0.17**	**0.861**	**.9637443**	**1.031371**
ln(Y)	1	(exposure)				

The men have approximately 1% less incidence of chewing difficulty than women across the entire dataset when adjusting for other effects. The p-value for sex term highlights that the effect for gender is not significant at the 5% level. This measure of significance, however, assumes that the effect of gender is proportional over both time scales and data of birth cohort.

Table S2. Estimated effects of chewing difficulty with “acp” parameterization of the APC model

apcfit, age(A) cases(D) poprisktime(Y) period(P) cohort(C) agefitted(agefitted) perfitted(perfitted) cohfitted(cohfitted) refper(2007) refcoh(1951) drextr(weighted) nper(100) param(**ACP**)

```
Iteration 0:   log likelihood = -12783.131
Iteration 1:   log likelihood = -12339.065
Iteration 2:   log likelihood = -12338.754
Iteration 3:   log likelihood = -12338.754

Generalized linear models                  No. of obs      =      2,151
Optimization     : ML                        Residual df     =      2,136
                                             Scale parameter =          1
Deviance        =  20289.19179              (1/df) Deviance =   9.498685
Pearson         =  16820.79616              (1/df) Pearson  =   7.874905

Variance function: V(u) = u                [Poisson]
Link function    : g(u) = ln(u)              [Log]

                                             AIC             =   11.48652
Log likelihood   = -12338.75412              BIC             =   3898.194
```

D	Coef.	OIM Std. Err.	z	P>|z|	[95% Conf.	Interval]
**_spA1_intct**	**-.6752659**	**.0657166**	**-10.28**	**0.000**	**-.804068**	**-.5464638**
_spA2	-.628401	.0706822	-8.89	0.000	-.7669356	-.4898664
_spA3	-.1513889	.0404979	-3.74	0.000	-.2307634	-.0720145
_spA4	.086676	.0271718	3.19	0.001	.0334203	.1399317
_spA5	.0410601	.0194547	2.11	0.035	.0029295	.0791906
_spA6	.0014715	.0133485	0.11	0.912	-.0246911	.0276342
_spP1	-.1291463	.0091487	-14.12	0.000	-.1470774	-.1112152
_spP2	.2362342	.0101063	23.38	0.000	.2164263	.2560421
_spP3	.0634694	.0085847	7.39	0.000	.0466438	.080295
_spP4	-.0096904	.0090732	-1.07	0.286	-.0274736	.0080928
**_spC1_ldrft**	**-.0637781**	**.0037851**	**-16.85**	**0.000**	**-.0711967**	**-.0563595**
_spC2	.1771702	.0383736	4.62	0.000	.1019594	.252381
_spC3	.0076103	.024672	0.31	0.758	-.0407459	.0559666
_spC4	.0019332	.0234965	0.08	0.934	-.0441192	.0479856
_spC5	-.0210841	.0211609	-1.00	0.319	-.0625587	.0203905
ln(Y)	1 (exposure)					

Akaike's information criterion and Bayesian information criterion

**Model**	**Obs**	**ll(null)**	**ll(model)**	**df**	**AIC**	**BIC**
**.**	**2,151**	**.**	**-12338.75**	**15**	**24707.51**	**24792.61**

Note: N=Obs used in calculating BIC; see [R] BIC note.

Table S3. Estimated effects of chewing difficulty with "apc" parameterization of the APC model

```
 apcfit, age(A) cases(D) poprisktime(Y) period(P) cohort(C) agefitted(agefitted)
perfitted(perfitted) cohfitted(cohfitted) refper(2007) refcoh(1951) drextr(weighted)
nper(100) param(APC)

Iteration 0:   log likelihood = -12783.138
Iteration 1:   log likelihood = -12339.071
Iteration 2:   log likelihood = -12338.761
Iteration 3:   log likelihood = -12338.761

Generalized linear models                  No. of obs      =      2,151
Optimization     : ML                        Residual df     =      2,136
                                             Scale parameter =          1
Deviance         =  20289.20488              (1/df) Deviance =   9.498691
Pearson          =  16820.81201              (1/df) Pearson  =   7.874912

Variance function: V(u) = u                  [Poisson]
Link function    : g(u) = ln(u)              [Log]

                                             AIC             =   11.48653
Log likelihood   = -12338.76066              BIC             =   3898.207

------------------------------------------------------------------------------
             |                 OIM
           D |      Coef.   Std. Err.      z    P>|z|     [95% Conf. Interval]
-------------+----------------------------------------------------------------
 _spA1_intct |  -2.472771    .059843   -41.32   0.000    -2.590061   -2.355481
       _spA2 |   .4761667   .0223741    21.28   0.000     .4323142    .5200192
       _spA3 |  -.1513869   .0404979    -3.74   0.000    -.2307612   -.0720125
       _spA4 |   .0866783   .0271718     3.19   0.001     .0334226    .1399339
       _spA5 |   .0410607   .0194547     2.11   0.035     .0029302    .0791913
       _spA6 |   .0014721   .0133485     0.11   0.912    -.0246906    .0276347
 _spP1_ldrft |  -.0637766   .0037851   -16.85   0.000    -.0711952   -.0563581
       _spP2 |  -.1291463   .0091487   -14.12   0.000    -.1470774   -.1112152
       _spP3 |   .2362342   .0101063    23.38   0.000     .2164263    .2560421
       _spP4 |   .0634693   .0085847     7.39   0.000     .0466437     .080295
       _spP5 |  -.0096904   .0090732    -1.07   0.286    -.0274736    .0080927
       _spC1 |   .1771676   .0383735     4.62   0.000     .1019569    .2523784
       _spC2 |   .0076121    .024672     0.31   0.758    -.0407441    .0559683
       _spC3 |   .0019345   .0234965     0.08   0.934    -.0441179    .0479868
       _spC4 |  -.0210832   .0211609    -1.00   0.319    -.0625577    .0203914
       ln(Y) |          1  (exposure)
------------------------------------------------------------------------------
```

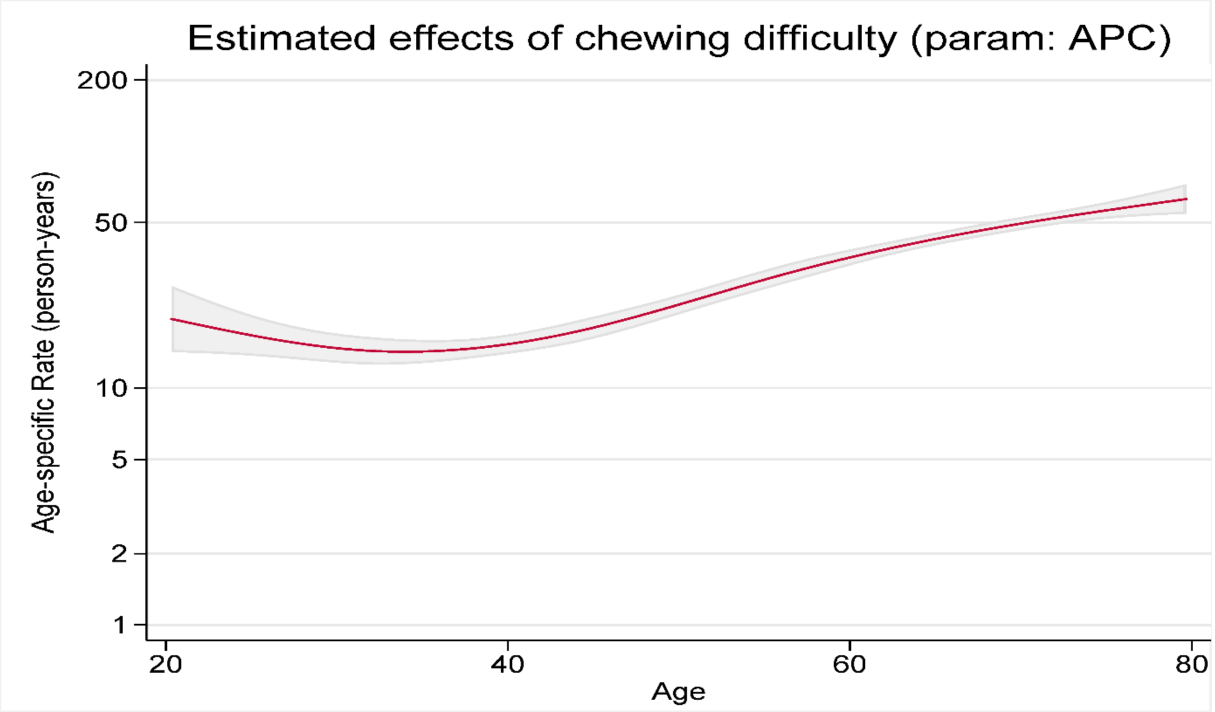

Figure S1. Longitudinal age curve illustrating the expected age-specific rates of chewing difficulty after adjusted for cohort effects for both genders combined.
